# Sexual dimorphism in the immune response in cardiovascular disease

**DOI:** 10.3389/fimmu.2026.2001502

**Published:** 2026-10-30

**Authors:** Mario Schubert, Friederike Lohse, Vasileia Ismini Alexaki

**Affiliations:** 1 Institute of Pharmacology and Toxicology, Faculty of Medicine, Technische Universität Dresden, Dresden, Germany; 2 Institute for Clinical Chemistry and Laboratory Medicine, Faculty of Medicine and University Hospital Carl Gustav Carus, Technische Universität Dresden, Dresden, Germany

**Keywords:** cardiovascular disease, lymphocytes, myeloid cells, sex chromosomes, sex hormones, sexual dimorphism

## Abstract

Cardiovascular disease (CVD) is a collective term for inflammatory pathologies including coronary artery disease and heart failure. Although sex-based differences in prevalence, progression and manifestation of CVD are well-established—mainly linked to hormonal status and Y-chromosome mosaicism—the exact role of the immune system in sexual dimorphism in CVD remains elusive. Immune responses differ in males and females and are primarily dictated by sex steroids and X-linked gene expression. Given the critical role of the immune system in CVD pathogenesis, we address the question, to what extent do these sexually dimorphic immune responses underpin the sex differences observed in CVD.

## 1 Introduction

The rising rates of obesity and its associated conditions—such as hypertension, dyslipidemia, and type 2 diabetes mellitus (T2DM)—have propelled cardiovascular disease (CVD) to pandemic levels. Although sex-based differences in CVD are extensively documented, the biological pathways driving this sexual dimorphism remain poorly elucidated (1). Structural or functional cardiac alterations occurring in asymptomatic individuals prior to overt cardiometabolic events exhibit sexual dimorphism (1). For example, women are more prone to left atrial dilation, whereas men more frequently present with left ventricular hypertrophy (1, 2). The impact of risk factors on the development of CVD also differs by sex (3, 4). T2DM confers a higher risk for coronary heart disease in women compared to men, which may stem from a chronically elevated CVD risk profile during the prediabetic state in women (5). On the other hand, men are more prone to abdominal fat deposition than women, which associates with increased risk for CVD (6). Atrial fibrillation is more prevalent in men and its risk rises with advancing age and increasing body mass index. However, when women develop atrial fibrillation, they face a higher risk of stroke, myocardial infarction (MI) and heart failure than their male counterparts (1, 7). After MI, men were reported to exhibit larger infarct sizes than women (8, 9) and worse short-term prognosis after first time MI (10). Regarding post-MI structural alterations, left ventricular wall thickening and cardiomyocyte hypertrophy is less pronounced in female patients, indicating reduced maladaptive remodeling and improved cardiac adaptation (11). In addition, women exhibit less cardiomyocyte cell death in the failing heart (12).

In all of these entities, immune cells and inflammatory processes are critically involved, as exemplified by their key role in tissue homeostasis and electrical conduction in the heart, and cardiac remodeling after MI (13–15). The key role of the immune response in the sexually dimorphic manifestation of CVD is exemplified by the higher prevalence of autoimmune and inflammatory conditions, such as rheumatoid arthritis and systemic lupus erythematosus (SLE) and their association with accelerated atherosclerosis in women (16). The present review illuminates the contribution of sex-specific immune responses to cardiovascular pathology given the established role of immune function in CVD and its marked sex dependence.

## 2 The role of immune cells in cardiovascular disease with particular focus on cardioimmunology

Both innate and adaptive immunity are key determinants in the pathogenesis of CVD. Elevated levels of inflammatory markers—including IL1β, C-reactive protein (CRP), serum amyloid A (SAA), IL6 and TNF—are predictive of increased CVD risk (17). In particular IL1β is a prognostic marker for severely reduced left ventricular ejection fraction (18). It also correlates with acute coronary syndrome and has diagnostic ability for major adverse cardiovascular events (19). Moreover, it is a strong and independent predictor of mortality in patients with idiopathic dilated cardiomyopathy and kidney transplant recipients (20, 21). Consequently, modulating immune responses has become a central focus in the development of therapeutic strategies for CVD prevention and treatment (17). The Canakinumab Anti-inflammatory Thrombosis Outcome Study (CANTOS) showed that targeting IL1β with the monoclonal antibody Canakinumab, significantly lowers the risk of recurrent cardiovascular events (22). In patients with diabetes and high vascular risk, canakinumab reduced circulating IL6 and CRP levels without affecting low-density lipoprotein (LDL) levels (23). CANTOS was a proof-of-concept study demonstrating the direct effects of inflammation on CVD without the involvement of an altered lipid profile. Moreover, the Colchicine Cardiovascular Outcomes Trial (COLCOT), in which patients received low-dose colchicine (0.5 mg once daily), demonstrated that suppressing inflammation in coronary atherosclerosis reduced ischemic cardiovascular events in patients with a prior MI (24). Dissecting the immune response to MI or other cardiovascular events is crucial for the development of effective therapeutic treatments.

In steady state conditions, the cardiac tissue contains tissue-resident macrophages (CCR2^-^) that develop from yolk sac and fetal liver progenitors. These are maintained through local proliferation and orchestrate coronary vascular maturation and growth (25–28). Both, MI and myocardial ischemia/reperfusion (MI/R) elicit an immune response triggered by damage-associated molecular patterns (DAMPs) released from dying cardiac cells (17). Upon MI, tissue resident CCR2^-^ cardiac macrophages are depleted and replaced by monocyte-derived pro-inflammatory CCR2^+^ macrophages (25, 29–32). Moreover, cardiac injury triggers recruitment of Ly6C^hi^ monocytes, which exhibit phagocytic, proteolytic and inflammatory functions, followed by expansion of reparative Ly6C^lo^ macrophages (25, 33, 34). Similarly, in the failing heart CCR2^+^ macrophages drive myocardial inflammation and heart failure pathogenesis, while tissue resident CCR2^-^ macrophages exert protection against dilated cardiomyopathy and impaired ventricular remodeling through their interaction with cardiomyocytes (35).

Neutrophils normally contribute to cardiac tissue homeostasis by preventing infections and promoting vascular renewal (17). Following MI, neutrophils are rapidly recruited to the injured myocardium and exacerbate inflammation via secretion of pro-inflammatory cytokines, chemokines, proteases, and the formation of neutrophil extracellular traps (NETs). In later stages of inflammation, neutrophil apoptosis and their subsequent clearance drives macrophage polarization toward a pro-resolving phenotype and promotes resolution of inflammation (17, 36).

Eosinophil numbers increase in the circulation and heart in mice post-MI, while their depletion exacerbates cardiac dysfunction, cell death and fibrosis. Accordingly, post-MI IL5 produced by group 2 innate lymphoid cells (ILC2s) promotes eosinophil and dendritic cell (DC) accumulation in the heart and facilitates recovery of cardiac dysfunction (37). The cardioprotective effect of eosinophils is mediated by IL4, a cytokine with pro-reparative and anti-inflammatory functions (37–39). However, IL4 may also promote pathological remodeling and fibrosis due to activation of cardiac CD206^+^IL4Rα^+^ macrophages in ischemic cardiomyopathy (40). In patients with acute MI, deregulated (either too high or too low) blood eosinophil counts represent a risk factor for cardiac deaths (41).

After MI, DCs infiltrate the heart and prime autoreactive T cells (42). Conventional type 2 DCs (cDC2s) present a-myosin heavy chain and induce the formation of Th1/Th17 autoreactive T cells (42). Plasmacytoid DCs (pDCs) are activated upon myocardial ischemia and produce type I interferon (IFN) exacerbating cardiac injury (43). In addition, cross-priming DCs activate cytotoxic CD8^+^ T cells contributing to myocardial damage (44). Natural Killer (NK) cells also infiltrate the infarcted myocardium in a CCR2-dependent manner, where they secrete granzyme B thereby exacerbating myocardial inflammation, promoting cardiomyocyte apoptosis and driving adverse ventricular remodeling, ultimately impairing cardiac function (45). Accordingly, administration of neutralizing antibodies targeting NK cells promote cardiac repair in a murine model of MI (45).

Early after the ischemic injury, CD4^+^ helper T cells are recruited to the myocardium, where they amplify inflammation and promote further immune cell infiltration (46, 47). Th1 cells enhance the inflammatory response by exacerbating cardiomyocyte death due to IFNγ signaling, while they also promote clearance of cellular debris and antifibrotic processes increasing the risk for cardiac rupture (48, 49). During the late phase of the post-MI inflammatory cascade, Th2 lymphocytes expand in number. These cells release type-2 cytokines (IL4, IL5, IL13), which suppress Th1-mediated responses and drive macrophages toward an alternative (M2-like) phenotype. This phenotypic switch away from classical (M1-like) activation ultimately supports tissue repair and promotes fibrotic remodeling (37, 49–51). However, while Th2 responses promote tissue healing, impaired Th2 responses may lead to pathological scarring and heart failure (52). Furthermore, Th17 cells infiltrate the ischemic heart and secrete IL17 that drives cardiomyocyte apoptosis, neutrophil infiltration and myocardial fibrosis (53). IL17 attenuates efferocytosis of Ly6C^hi^ monocyte-derived macrophages by inducing GM-CSF production from cardiac fibroblasts (33). Th17 cells also secrete IL21 that impedes the healing process by promoting apoptosis of Ly6C^lo^ macrophages and increasing neutrophil infiltration (54). Patients with chronic heart failure present an imbalanced Th17/regulatory T cell (Treg) ratio (55). Tregs modulate the cardiac immune response following MI by reducing CD8^+^ T cells, modulating macrophage activation, as well as exerting direct pro-regenerative effects in non-immune cells such as fibroblasts, endothelial cels and cardiomyocytes limiting cardiomyocyte death and reducing fibrosis (56–60). Exogenously applied Tregs are rapidly recruited to the infarcted heart and lead to reduction of CCR2^+^Ly6C^hi^ monocytes/macrophages and increase of a pro-resolving macrophage phenotype through an IL10-dependent mechanism (61).

CD8^+^ T cells are rapidly recruited to the ischemic cardiac tissue (47). There they amplify inflammation due to their cytotoxicity to cardiomyocytes, yet they also promote macrophage-mediated clearance of necrotic tissue and mitigate cardiac fibrosis (62, 63). Hence, CD8^+^ T cells have both beneficial and detrimental roles in cardiac remodeling (62). Preclinical models demonstrate that pharmacological blockade of T-cell co-stimulation curtails both T-cell infiltration and activation, consequently reducing tissue injury and cardiac dysfunction in murine heart failure (64). The presence of cardiac-specific autoantibodies in heart failure patients further implicates the humoral arm of autoimmunity in worsening myocardial dysfunction (65).

Finally, B cells are recruited to the injured myocardium, where they can promote inflammation and fibrosis (66, 67). After acute MI in mice, mature B cells produce CCL7 and induce Ly6C^hi^ monocyte recruitment to the heart promoting tissue injury and myocardial dysfunction (68). Accordingly, high blood concentration of CCL7 in patients with acute MI associates with increased risk of death or recurrent MI (68).

## 3 Sex dimorphism in the immune system

The immune system differs considerably between males and females (69). Men have larger numbers of CD14^+^ (classical) and CD16^+^ (non-classical) monocytes and overall higher proportion of myeloid cells, as well as larger proportion of activated NK cells (69, 70). Women exhibit higher CD4^+^ T-helper counts and a higher CD4^+^/CD8^+^ T-cell ratio than age-matched men, whereas men have higher proportion of CD8^+^ T cells (69, 70). Women also have higher B cell and plasma cell counts and higher immunoglobulin levels (69, 70). Moreover, women have greater numbers of CD25^hi^CD127^lo^ Tregs and mucosa-associated invariant T cells (MAIT) (71). DC numbers do not substantially differ between men and women, but pDCs from women produce more IFNα following Toll-Like Receptor 7 (TLR7) and TLR8 ligand stimulation compared to pDCs from men (69, 72–74). In general, women and female mice have a more modest response to inflammatory stimuli; for example, in sepsis and atherosclerosis, they have lower pro-inflammatory leukocyte-mediated inflammation and accelerated resolution of inflammation compared to male counterparts (75, 76).

Single-cell RNA sequencing (scRNA-seq) in peripheral blood mononuclear cells (PBMCs) revealed an increased expression of genes related to oxidative stress and inflammation, such as DNA-damage-inducible transcript 4 (*DDIT4*), *HLA-DRB5*, zinc finger protein 36 (*ZFP36*), *JUNB*, and C-X-C chemokine receptor type 4 (*CXCR4*), across multiple immune cell populations, such as NK cells and monocytes, in healthy elderly men compared to age-matched healthy women (70). However, following vaccination in adult humans or viral infection in rats, the expression of genes related to type I IFN response such as *TLR7*, Retinoic acid-inducible gene I (*RIGI*), Interferon Regulatory Factor 7 (*IRF7*), *IFNB* or Signal Transducer And Activator Of Transcription 3 (*STAT3*) is higher in female than male human PBMCs or rat tissues (69, 77). Upon TLR9 activation, PBMCs from men respond with stronger IL10 production compared to PBMCs from women (78). Male PBMCs also produce more TNF than female PBMCs upon lipopolysaccharide (LPS) stimulation (69). Moreover, neutrophils from men express higher levels of TLR4 and produce more TNF than human female neutrophils both constitutively and following activation with LPS (79). In turn, phagocytic activity of neutrophils and macrophages as well as the antigen presentation is more efficient in female than male mice, and female mice eliminate more efficiently bacteria in a sepsis model due to more efficient clearance of systemic infections by liver macrophages (69, 76, 80). Aging increases inflammatory activation of myeloid cells, including TNF, IFNγ and IL1 pathways, more substantially in men than women (81). Overall, genomic differences between sexes increase in advanced age with men having higher innate and pro-inflammatory activity and lower adaptive immunity (69, 81).

Inflammation is a strong driver of coronary artery disease (CAD), a common heart disease, where atherosclerotic plaque formation narrows the coronary arteries and reduces blood flow to the cardiac muscle. Monocytes and macrophages accumulate in the plaques and promote plaque formation, progression and rupture (82). Monocytes from female coronary artery disease patients exhibit a transcriptional profile marked by a stronger chemotaxis signature and elevated expression of type I IFN, granulocyte-macrophage colony-stimulating factor (GM-CSF), CD40L, epidermal growth factor (EGF), and vascular endothelial growth factor (VEGF) compared to their male counterparts (83). In contrast, monocytes from male patients show upregulation of IL4 and High mobility group box 1 (HMGB1) signaling. These sex-based transcriptional differences are absent in healthy individuals (83). Following MI, male mice exhibit both a greater infarct expansion index, elevated incidence of cardiac rupture, higher cardiac dilatation and higher mortality rates (84, 85). Female mice post-MI show less left ventricular dilatation, reduced pathological hypertrophy, better preserved systolic function, and smaller infarct sizes (86, 87). Female mice also show increased tolerance to ischemia reperfusion injury, reduced fibrotic remodeling and lower infiltration of proinflammatory monocytes compared to male mice (88). Accordingly, post-MI female mice have fewer yet more efficient tissue clearing neutrophils with reduced pro-inflammatory gene expression (87). Neutrophils from female mice rely on thrombospondin-1 stimulation of CD36 to promote wound healing (87). After permanent coronary ligation in mice, females exhibit higher immune T and B cell infiltration in the heart (89). Female mice also show heightened expression of *Il6*, *Ifng*, *Tnf*, *Ccl2* and *Il10* in the infarcted and remote tissue, and increased spleen mass alongside with higher proinflammatory cytokine levels in the spleen (89). Moreover, IgG and IgM deposition in the cardiac tissue, plasma IgM and plasma autoantibodies are more pronounced in female mice, suggesting that MI-induced systemic and myocardial autoimmune activation is stronger in females (89) (**Figure 1**). Sex-dependent differences in the MI-induced immune responses are not restricted in the heart. After left anterior descending (LAD) artery ligation, male mice exhibit increases in TCRγδ^+^ T cells, while female mice show increased accumulation of neutrophils and Ly6C^hi^ monocytes in the small intestine associated with sex-dependent changes in the gut microbiome and metabolome (90).

**Figure 1:**
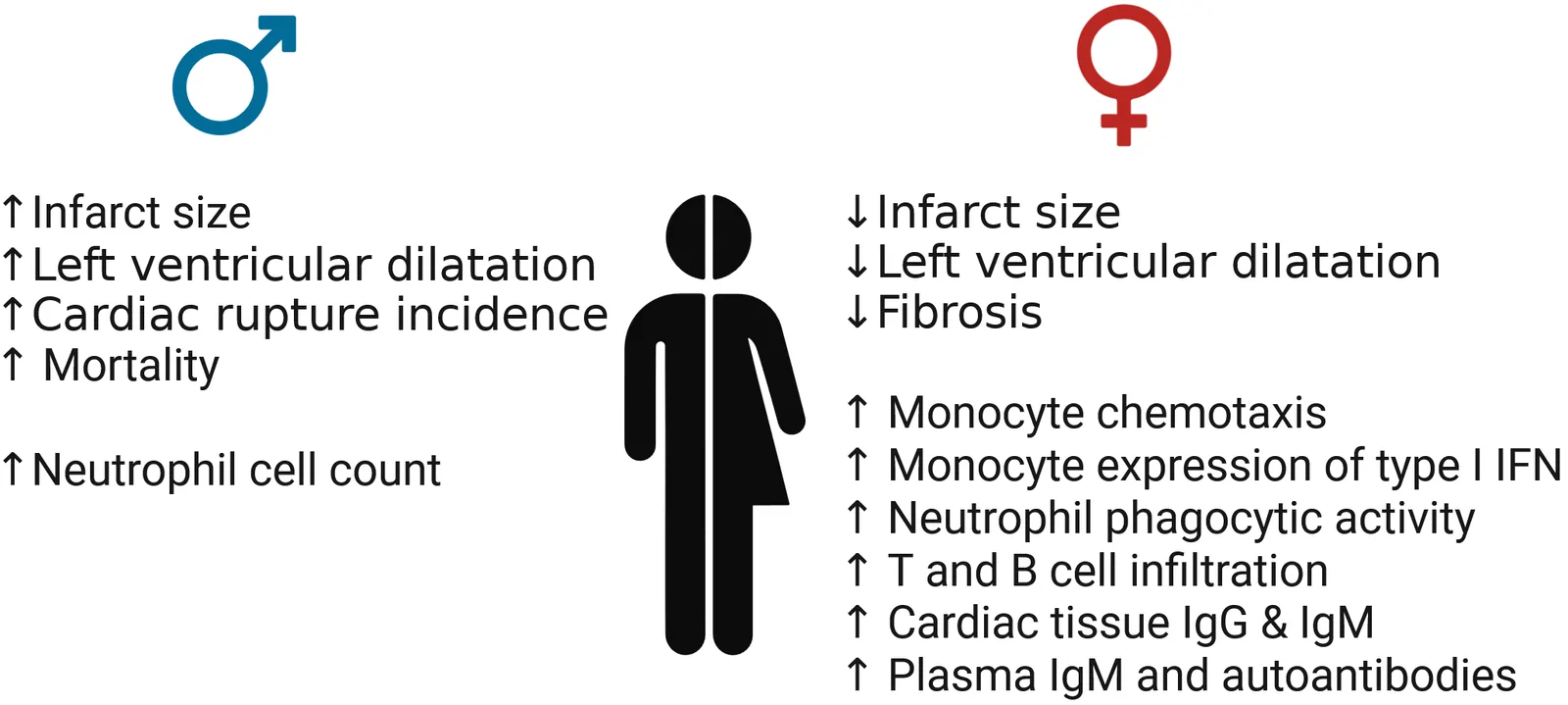
Sex dimorphism in the immune response after myocardial infarction. After reperfusion of a first-time MI, male patients typically show higher blood neutrophil counts than women (75). In female patients with coronary artery disease, monocytes display a stronger transcriptional signature of chemotaxis and increased type I IFN expression (83). In mice, males exhibit greater infarct expansion index, higher incidence of cardiac rupture, more cardiac dilatation, and higher mortality than females (84, 85). In contrast, female mice have fewer but more efficient tissue-clearing neutrophils (87), greater T and B cell infiltration, enhanced IgG and IgM deposition in cardiac tissue, higher plasma IgM and autoantibody levels, and attenuated fibrosis (88, 89).

## 4 The role of sex chromosomes in the immune responses in cardiovascular disease

### 4.1 Y chromosome

The Y chromosome is small and largely noncoding with around 70 protein-coding genes (91). Yet, it is Y chromosome abnormalities that are linked to cardiovascular risk, while the role of the X chromosome in CVD remains unclear (73). The incidence of hematopoietic mosaic loss of the Y chromosome (mLoY) rises markedly with advancing age in men and is associated with elevated risks of CVD, atrial fibrillation, heart failure and death by cardiovascular causes (73, 92–98). Single-cell RNA-seq analysis of PBMCs from ischemic cardiomyopathy patients identified mLoY predominantly in monocytes associated with a pro-inflammatory and pro-fibrotic transcriptional profile, demonstrated by increased expression of inflammatory markers (S100 calcium-binding protein A8 (*S100A8*), *TLR2*, C-type lectin receptor (*CLEC4D*)) and decreased expression of TGF-β inhibitors (*SMAD7*, *TGIF2*) (92) (**Figure 2**). In patients with both mLoY and DNA-methyltransferase 3A (*DNMT3A*) mutations - the latter driving clonal hematopoiesis of indeterminate potential (CHIP) - monocytes show enhanced expression of pro-inflammatory genes, including alarmins (*S100A8*, *HMGB2*) and IFN signaling-related genes (*IFNGR1*, *TRIM56*, *CD84*) (92). These findings are consistent with other studies, which demonstrate a pro-fibrotic phenotype in monocytes with mLoY from patients with aortic stenosis (99) and a positive correlation between the proportion of cardiac macrophages with mLoY and fibroblast activation in cardiac biopsy samples from patients with dilated cardiomyopathy (100).

**Figure 2:**
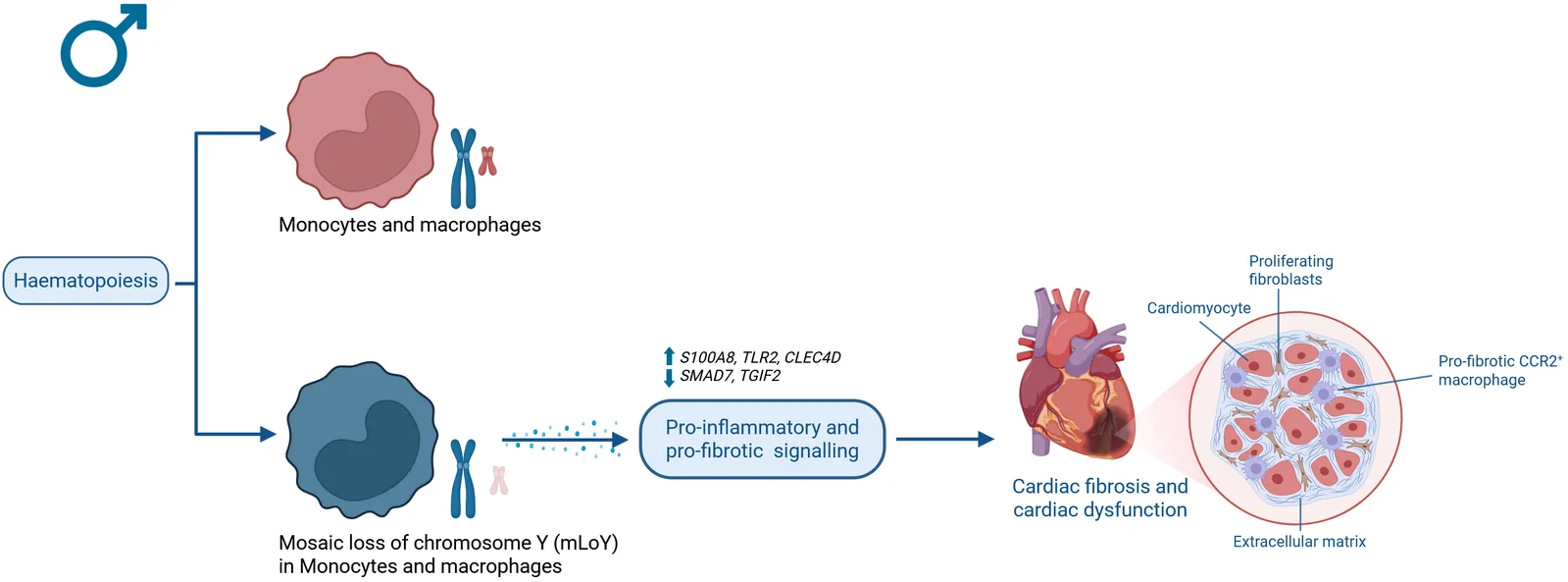
The role of mLoY in immune cells in cardiomyopathy. In patients with ischemic cardiomyopathy, monocyte mLoY is associated with a pro-inflammatory and pro-fibrotic transcriptional profile, characterized by increased expression of *S100A8*, *TLR2* and *CLEC4D* and decreased expression of *SMAD7* and *TGIF2* (92). In rodents, mLoY leads to greater cardiac fibrosis and dysfunction in response to pressure overload induced by transaortic constriction (98, 100).

A causal role of mLoY in CVD was also observed in mouse models of mLoY after bone marrow reconstitution with cells lacking the Y chromosome. Animals with mLoY developed exacerbated cardiac fibrosis and cardiac dysfunction in response to pressure overload by transaortic constriction, accompanied by a greater abundance of CCR2^+^ cardiac macrophages with a pro-fibrotic (indicated by upregulated TGF- β signaling) but less inflammatory (shown by downregulated IL1β signaling) gene expression profile (98) (**Figure 2**). The Y-chromosomal gene ubiquitously transcribed tetratricopeptide repeat containing, Y-linked (*Uty*), encoding a histone demethylase involved in the epigenetic regulation in hematopoietic stem cells (HSCs), is key in protection against heart failure progression in mice (100). Disruption of *Uty* in HSCs, as present in mLoY, accelerates cardiac dysfunction after transaortic constriction and is associated with a more pro-fibrotic gene expression signature in macrophages and increased fibroblast proliferation (100). Notably, blockade of neutrophil- and monocyte infiltration using a granulocyte receptor-1 antibody normalized fibroblast abundance and attenuated the decrease in cardiac function, confirming that altered phenotype of myeloid cells in mLoY drives the development of cardiac fibrosis and dysfunction (98).

### 4.2 X chromosome

The X chromosome ranks among the largest chromosomes and contains around 850 protein-coding genes (74). Aortopathies, such as aortic aneurysms or aortic dissections, have lower prevalence in women (46, XX) compared to males (46, XY) and Turner syndrome individuals (45, XO), suggesting a protective role of the X chromosome (101, 102). However, mouse models revealed that both, a lower copy number of X chromosomal genes but also treatment with testosterone increases the incidence of aneurysms (103, 104). Therefore, it remains unclear whether the lower incidence of aneurysms in women are solely due to higher X chromosome dosage or also due to low testosterone levels (102, 104). In addition to inherited chromosomal aneuploidies, mosaic loss of the X chromosome (mLoX) is detected in a small fraction (~2%) of leukocytes from ~12% of female individuals (105). However, no significant associations between mLoX and CVD were found so far (73, 94).

The X chromosome carries several genes with immune functions, including genes that are important for immune cell identity like *FOXP3*, cellular activation and signalling triggered by immune signals i.e. *CD40LG*, *TLR7*, Interleukin-1 receptor-associated kinase 1 (*IRAK1*), *IL13RA1/2* or *NEMO*, leukocyte trafficking (*CD99* and *CXCR3*), or immune cell differentiation and proliferation (*IL2RG* and Bruton tyrosine kinase (*BTK*)) (69, 74). In female mammals, one X chromosome is transcriptionally silenced (XCI) through the long non-coding RNA X-inactive specific transcript (*Xist*) and repressive histone modifications, which limit the expression of X-chromosomal genes to one allele (74). However, approximately 15–23% of X-linked genes in humans ‘escape’ XCI either constitutively or in a cell-type-specific manner and are transcribed from the inactive X chromosome (Xi) (74). Interestingly, pDCs lack *Xist* RNA localization at the Xi and escape from XCI (106, 107). XCI escape for the *TLR7* gene underlies stronger type I IFN responses in pDCs of females (106, 107). Similarly, the DEAD-Box Helicase 3 X-Linked (*DDX3X*) gene also escapes XCI in pDCs and promotes type I IFN-induced immune responses (108). In contrast, NK cells have *Xist* RNA transcripts dispersed across the nucleus and bone marrow derived macrophages (BMDMs) have *Xist* RNA clusters at the Xi together with the heterochromatin mark H3K27me3 (106). XCI escape of *Kdm6a*, encoding the protein UTX, underlies stronger effector function of NK cells in female mice (109). Hence, XCI escape renders the chromatin of the Xi in female immune cells more euchromatic than in male counterparts, and therefore prone to aberrant reactivation at certain loci. This mechanism may potentiate the expression of X-linked genes in response to pathogen challenge or tissue injury and predispose to autoimmunity (110). As a result, women typically mount a more robust immune response and achieve faster clearance of the insult than men—a difference that may influence both the development and clinical trajectory of CVD. However, this enhanced immunological reactivity comes at a cost, predisposing females to autoimmune conditions, which disproportionately affect women (69). Notably, individuals with autoimmune diseases such as SLE or rheumatoid arthritis face a substantially elevated risk of CVD compared to their healthy counterparts (111, 112). Taken together, although still elusive, the role of XCI escape in CVD holds promise as an explanation for sex differences in CVD (73).

XCI skewing is common in carotid plaques and correlates with peripheral artery events in female patients (113). scRNA-seq analyses identified marked sex differences in macrophage states in atherosclerotic plaques of carotid arteries. In men, unstable plaques were characterized by a depletion of contractile vascular smooth muscle cells (VSMCs), accompanied by an accumulation of inflammatory macrophages and activated naive T lymphocytes, while in women unstable plaques retained a higher proportion of contractile VSMCs but displayed a more pronounced expansion of cytotoxic T lymphocytes, foam-cell macrophages and enhanced macrophage-macrophage interactions (114, 115). Sex-biased expression of X-linked genes was found in blood from acute ischemic stroke patients (116) and myocardial tissue from dilated cardiomyopathy patients (117, 118). XCI escape was also suggested to regulate sex differences in myofibroblast activation and subsequent aortic valve stenosis progression (119).

Another, yet largely unexplored, mechanism by which X-chromosomal genes may contribute to sexually dimorphic immune responses and CVD are monogenic mutations or variants in X-chromosomal genes. The phenotypic consequences caused by monogenic X-chromosomal variants are often more pronounced in males because of compensatory effects by the second, intact allele in females. For instance, TRL8 gain-of-function variants were associated with lymphoproliferation, neutropenia, infectious susceptibility, B- and T-cell defects, elevated serum levels of pro-inflammatory cytokines, and an enhanced inflammatory response of patient-derived macrophages and neutrophils generated from induced pluripotent stem cells (iPSCs) to TLR8 stimulation (120). Conversely, PBMCs from patients with severe COVID-19 carrying TLR7 loss-of-function variants exhibit impaired IFN signaling responses (121). Mutations in several X-chromosomal genes—including *DMD* (encoding dystrophin) in dilated cardiomyopathy, *KCNE5* (encoding a potassium channel subunit) in arrhythmias and Brugada syndrome, and *TAZ* (encoding the acyltransferase taffazin) in Barth syndrome—have been linked to these cardiac pathologies (73). However, to date, these mutations have not been directly implicated in modulating immune responses.

## 5 The role of sex hormones in the immune responses in cardiovascular disease

Sex hormone levels fluctuate substantially across the lifespan. In women, estradiol and progesterone levels vary with the menstrual cycle, rise sharply during pregnancy and decline after menopause, while testosterone levels are much higher in men than women and decline with aging (69). The role of sex hormones in CVD has been extensively addressed yet the underlying mechanisms remain largely unclear (122, 123). Low endogenous testosterone and estradiol levels in men or estrogen in women may be a CVD risk factor (122, 124). However, evidence regarding the impact of hormone therapy on CVD remains inconclusive (123, 125–127).

As mentioned above, female mice post-MI show smaller infarct sizes, while ovariectomized rodents develop larger infarcts and more severe I/R-induced dysfunction than males—effects that are partially rescued by chronic estrogen supplementation (86, 87, 128). Aging past menopause in female mice abrogates the protection against excessive IL6 signaling in younger females, an effect attributed to reduced liver X receptors/retinoid X receptor LXR/RXR activation (87).

Blood neutrophil numbers are higher in men than women after reperfusion of first-time MI, while tocilizumab, an interleukin-6 receptor inhibitor, reduces blood neutrophil counts and infarct size to a greater extent in men than women (85). Similarly, circulating neutrophil counts post-MI are higher in male than female mice, while castration-induced testosterone lack attenuates blood neutrophil counts to the level in female animals and increases survival post-MI (85). These effects are replicated in mice with genetic depletion of the androgen receptor in the bone marrow (85) (**Figure 3**). Moreover, testosterone deficiency through castration increases atherogenesis, an effect which is abolished with testosterone supplementation (129). Accordingly, androgen receptor deficiency results in an increased atherosclerotic burden in male mice (129). scRNA-seq in atherosclerotic plaques showed that elevated transcriptional activity in women is associated with mesenchymal and endothelial cells, whereas in men stronger transcriptional activity is associated with the immune system (130). Men with androgen deficiency have increased levels of pro-inflammatory cytokines (IL1β, TNF), decreased Treg and higher NK cell count, as well as increased antibody titers and CD4/CD8 T cell ratios compared to men with normal testosterone levels (69, 131). Comparison of neutrophils from female and male individuals with Klinefelter syndrome (47, XXY genotype) suggests that transcriptional differences related to the innate immune response, for instance including type I IFN signaling, are driven by estrogen and not X-chromosomal dosage (132). Neutrophil infiltration at the infarct border is more pronounced in male than female rodents during the first days of MI accompanied by increased matrix metalloproteinase (MMP) activity, while later on (4 days post-MI) the number of infiltrating macrophages is significantly higher in female animals (84). Treatment with 17β-estradiol attenuates cardiac fibroblast proliferation, collagen deposition and MMP activation through regulation of TNF production by cardiac immune cells (133). In rat models of MI/R, estrogen improves myocardial functional recovery and downregulates TNF, IL1β and IL6 (134, 135) (**Figure 3**).

**Figure 3:**
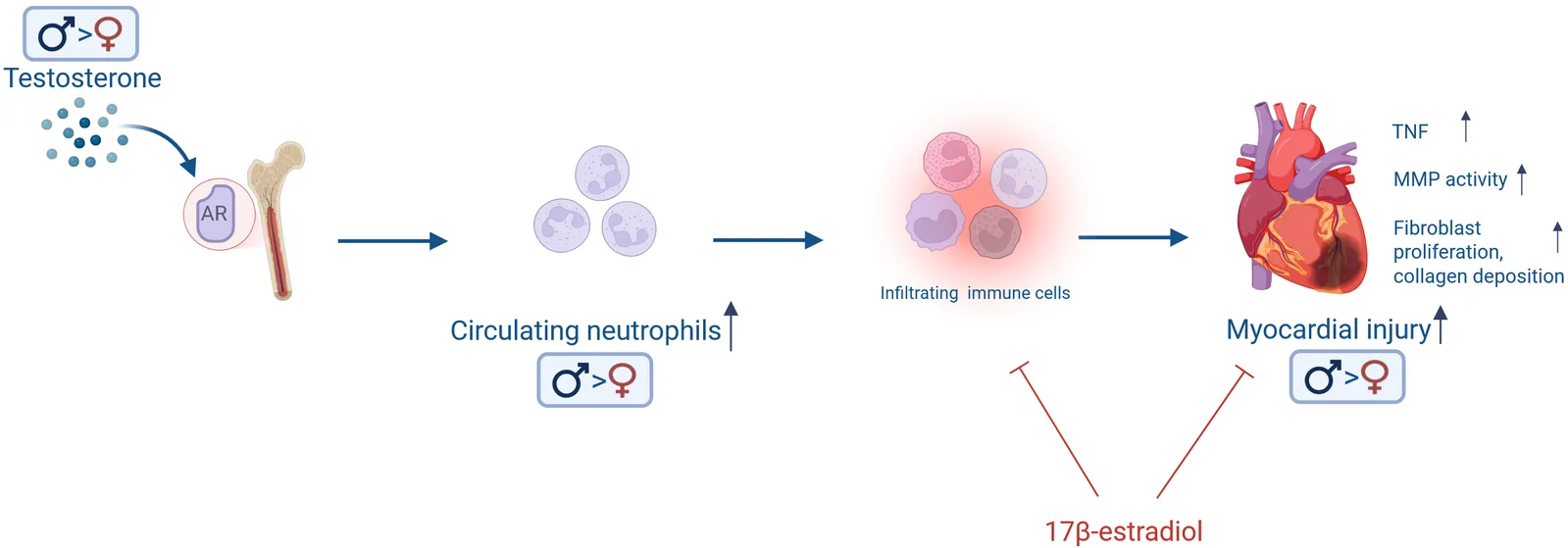
Sex hormones regulate the MI-induced immune response. After MI, circulating neutrophil counts are higher in male than female mice, whereas castration or genetic depletion of the androgen receptor in the bone marrow reduces blood neutrophil counts and improves survival (85). Consistent with this, neutrophil infiltration into the infarct area is more pronounced in male rodents (84). Treatment with 17β-estradiol limits cardiac fibroblast proliferation, collagen deposition, and MMP activation by regulating TNF production by cardiac immune cells (133). In rat models of MI/R, estrogen improves myocardial functional recovery and downregulates TNF, IL1β, and IL6 (134, 135).

The effects of sex hormones in immune cells have been extensively described (69, 131). Both lymphocytes and myeloid cells express classical estrogen receptor alpha (ERα) and ERβ, which mediate genomic effects of estrogen (131). Estradiol is thought to have a bipotential function: at low doses enhancing proinflammatory activation of monocytes/macrophages and DCs, antigen presentation and Th1 responses, while in high doses reducing proinflammatory responses and augmenting Th2 responses and humoral immunity (69, 131). In addition, estradiol signals through the non-classical G protein-coupled estrogen receptor 1 (GPER1) regulating neutrophil apoptosis, mitigating the inflammatory response in monocytes/macrophages, increasing IL10 production in CD4^+^ T cells and regulating B cell proliferation (136). Progesterone exerts broad anti-inflammatory effects in macrophages, DCs and NK cells, while skewing CD4^+^ T cell responses from Th1 to Th2-type responses and increasing the Treg/Th17 ratio (69, 131). Testosterone and dihydrotestosterone also have overall immunosuppressive function inhibiting the proinflammatory activity of macrophages and NK cells and promoting anti-inflammatory mechanisms (69, 131).

Finally, estrogen may also determine CVD due to regulation of clonal hematopoiesis. *DNMT3A* clonal hematopoiesis associates with MI in women but not men (137). Accordingly, hematopoietic *DNMT3A* mutations are more common in women (138). HSCs with *Dnmt3a* mutation from female mice maintain stemness and show a higher proliferation rate in response to estrogen compared to wild-type HSCs, which likely contributes to the predominant prevalence of *DNMT3A* mutation-related clonal hematopoiesis in females (138). Hence, estrogen may increase the risk for MI in women by promoting *DNMT3A*-mutant clonal hematopoiesis (137, 138).

## 6 Perspectives and future directions

The integration of multiple publicly available proteomic, metabolomic and transcriptomic datasets and the increasing availability of such datasets with single-cell and spatial resolution, holds great potential to elucidate the mechanisms underlying sex dimorphism in cardiovascular disease and the role of the immune system therein (139). Furthermore, investigating sex dimorphism in systemic molecular dynamics of interorgan crosstalk in cardiometabolic disease, will deepen our understanding of cardioimmunology and pathophysiology of cardiovascular disease (140). With respect to human-relevant experimental models, iPSCs hold enormous potential for studying inherent cellular differences between sexes due to their donor- or patient-specific nature. This is particularly relevant for identifying the mechanisms underlying disease development, as iPSCs enable the generation of (healthy) human cell types, including atrial and ventricular cardiomyocytes (141), cardiac fibroblasts (142), cardiac endothelial cells (143), and macrophages (iMacs) (144). For instance, recent analyses of male and female iMacs revealed diverging inflammatory responses to LPS treatment (145). In addition, human iPSC-based cardiac models recapitulating key features of HFpEF were recently established that could be used to investigate sex dimorphism and the role of the immune system in HFpEF (146, 147). Moreover, sex-stratified analysis should become a mandatory prespecified endpoint in immunomodulatory cardiovascular trials, not a *post-hoc* exploration. The aforementioned technologies and approaches will provide insights that should drive a shift from “sex-neutral” to “sex-responsive” trial designs to increase the probability of detecting differential treatment responses. Finally, most research or clinical studies currently treat sex as a binary variable. However, sex should not be implemented in clinical study design as a fixed dichotomy but as a spectrum of differential physiological states influenced by dynamic hormonal transitions due to aging, pregnancy, menopause or hormone therapy. This is particularly important in cardioimmunology and CVD, given that sex hormones strongly influence the immune responses (69, 131).

## 7 Conclusion

CVD is shaped by sex and the immune response. mLoY is associated with enhanced CVD and MI risk, alongside a pro-inflammatory shift of monocytes and a pro-fibrotic phenotype in cardiac macrophages (73, 92, 100). XCI escape - a key driver of sex-based immunological differences - renders women more responsive to pro-inflammatory stimuli (69, 74, 110). Moreover, testosterone promotes the expansion of circulating myeloid cells, while estrogen may help limit post-MI myocardial fibrosis. Despite these insights, a deeper, multi-level mechanistic understanding of sex dimorphism in the MI-induced immune response—one that spans from hematopoietic lineage commitment (myelopoiesis versus lymphopoiesis) and immune cell mobilization, through the regulation of inflammatory onset and resolution, to fibrosis and tissue repair - is needed. A deeper understanding of these sex-specific immune mechanisms could be leveraged to develop targeted therapies ultimately improving CVD outcomes for both men and women.
